# Prevalence of asthma, aspirin sensitivity and allergy in chronic rhinosinusitis: data from the UK National Chronic Rhinosinusitis Epidemiology Study

**DOI:** 10.1186/s12931-018-0823-y

**Published:** 2018-06-27

**Authors:** Carl M. Philpott, Sally Erskine, Claire Hopkins, Nirmal Kumar, Shahram Anari, Naveed Kara, Sankalp Sunkaraneni, Jaydip Ray, Allan Clark, Andrew Wilson, On behalf of the CRES group, Sally Erskine, Carl Philpott, Allan Clark, Claire Hopkins, Alasdair Robertson, Shahzada Ahmed, Naveed Kara, Sean Carrie, Vishnu Sunkaraneni, Jaydip Ray, Shahram Anari, Paul Jervis, Jaan Panesaar, Amir Farboud, Nirmal Kumar, Russell Cathcart, Robert Almeyda, Hisham Khalil, Peter Prinsley, Nicolas Mansell, Mahmoud Salam, Jonathan Hobson, Jane Woods, Emma Coombes

**Affiliations:** 1 James Paget University Hospital NHS Foundation Trust, Gorleston, UK; 2 Rhinology and Olfactology, University of East Anglia, Norwich, UK; 3 Guys and St Thomas’ Hospital, London, UK; 4 Wrightington, Wigan and Leigh NHS Foundation Trust, Wigan, UK; 5 Heart of England NHS Foundation Trust, Birmingham, UK; 6 Sunderland Royal Infirmary, Sunderland, UK; 7 Royal Surrey County Hospital, Guildford, UK; 8 Sheffield Teaching Hospitals, Sheffield, UK; 9 Norwich Medical School, University of East Anglia, Norfolk, NR4 7TJ UK

**Keywords:** Rhinitis, Sinusitis, Rhinosinusitis, Quality of life, Epidemiology, Asthma, Allergy, Aspirin sensitivity

## Abstract

**Background:**

Chronic rhinosinusitis (CRS) is a common disorder associated with other respiratory tract diseases such as asthma and inhalant allergy. However, the prevalence of these co-morbidities varies considerably in the existing medical literature and by phenotype of CRS studied. The study objective was to identify the prevalence of asthma, inhalant allergy and aspirin sensitivity in CRS patients referred to secondary care and establish any differences between CRS phenotypes.

**Methods:**

All participants were diagnosed in secondary care according to international guidelines and invited to complete a questionnaire including details of co-morbidities and allergies. Data were analysed for differences between controls and CRS participants and between phenotypes using chi-squared tests.

**Results:**

The final analysis included 1470 study participants: 221 controls, 553 CRS without nasal polyps (CRSsNPs), 651 CRS with nasal polyps (CRSwNPs) and 45 allergic fungal rhinosinusitis (AFRS). The prevalence of asthma was 9.95, 21.16, 46.9 and 73.3% respectively. The prevalence of self-reported confirmed inhalant allergy was 13.1, 20.3, 31.0 and 33.3% respectively; house dust mite allergy was significantly higher in CRSwNPs (16%) compared to CRSsNPs (9%, *p* < 0.001). The prevalence of self- reported aspirin sensitivity was 2.26, 3.25, 9.61 and 40% respectively. The odds ratio for aspirin sensitivity amongst those with AFRS was 28.8 (CIs 9.9, 83.8) *p* < 0.001.

**Conclusions:**

The prevalence of asthma and allergy in CRS varies by phenoytype, with CRSwNPs and AFRS having a stronger association with both. Aspirin sensitivity has a highly significant association with AFRS. All of these comorbidities are significantly more prevalent than in non-CRS controls and strengthen the need for a more individualised approach to the combined airway.

## Background

Chronic rhinosinusitis (CRS) is the term used to denote a common symptom set lasting for more than 12 weeks and requires endoscopic or radiological confirmation [1]. Such symptoms include nasal blockage, rhinorrhoea, facial pain and loss of sense of smell. CRS affects a significant proportion of the adult population with a recent European study suggesting a prevalence of 11% [2]. The pathophysiology for CRS is not yet fully understood but it is currently accepted to roughly divide cases into two common phenotypes – those with polyps and those without nasal polyps (CRSwNPs and CRSsNPs respectively). There are many proposed aetiological factors; viruses, bacteria and fungi alongside host and environmental factors have all been implicated with the likelihood of an array of underlying endotypes.

Allergic fungal rhinosinusitis (AFRS) is an increasingly recognised distinct subtype of CRSwNPs that represents a therapeutically more challenging condition. AFRS was first described in 1976 [3] where resected nasal mucosa from group of young adults with a history of asthma and chronic nasal polyps was found to contain similar histological features, including a distinct mucinous material containing eosinophils, Charcot-Leyden crystals, and fungal hyphae. The most commonly used classification today is that defined by Bent and Kuhn in 1994 [4] which states that AFRS is a condition associated with five major diagnostic criteria; 1) evidence of type I hypersensitivity (IgE mediated), 2) nasal polyposis, 3) characteristic computed tomography findings, 4) eosinophilic mucus, and 5) positive fungal smear, and six associated supporting criteria; 1) asthma, 2) unilateral predominance, 3) radiographic bone erosion, 4) fungal culture, 5) Charcot-Leyden crystals, and 6) serum eosinophilia.

In addition to the potential causative factors already described, aspirin is also known to exacerbate nasal symptoms. In some patients, this is as part of aspirin exacerbated respiratory disease (AERD) [5]. This was first described in 1922 by Widal [6] as a triad of symptoms including aspirin sensitivity, asthma, and nasal polyposis, more commonly known as Samter’s triad [7]. AERD initially includes upper airway symptoms such as nasal obstruction/congestion and rhinorrhoea, and progresses over months and years to development of lower airway symptoms, including shortness of breath, which can develop into life-threatening asthma [8].

The role of atopy in CRS is widely debated in the medical literature but it is generally accepted that it is not a definitive aetiological factor. The reports of the prevalence of allergic rhinitis in CRS vary wildly, ranging from as low as 10% to as high as 84% [9–13], with differing phenotype cases included in the relevant studies likely to be an influential factor. The European Position Paper on Rhinosinusitis and Nasal Polyps suggests that a selection bias in these studies by physicians with an interest in allergy, has led to artificially high reporting of inhalant allergy in CRS [1].

The prevalence of asthma in CRS also varies considerably in the literature, ranging from 4 to 44% [14–21], influenced by study design and phenotypes. The association between the two conditions is commonly recognised [22] and although the interaction is yet to be fully understood [23], recent early biomarker research suggests that higher serum periostin levels may denote cases of CRSwNPs with comorbid asthma [24]. It is certainly clear that both severity and duration of CRS are associated with increasing levels of comorbid asthma [25], suggesting poor control of CRS heralds more lower respiratory tract disease.

The overarching aims of the Chronic Rhinosinusitis Epidemiology Study (CRES) were to identify differences in socio-economic variables, medical co-morbidities and environmental exposures between patients with CRS and healthy controls. The aim of this specific analysis was to identify the prevalence of asthma, inhalant allergy and aspirin sensitivity in CRS patients referred to secondary care, and to establish any differences between CRS phenotypes and compared to control subjects without CRS. This data will help to inform NHS policy makers and clinical commissioning groups regarding their approach to upper and lower airway disease.

## Methods

The CRES was approved by the Oxford C Research Ethics Committee (Ref: 07/H0606/100), sponsored by the University of East Anglia (UEA) and funded by the Anthony Long and Bernice Bibby Trusts. Details of the full methods used for the whole study can be seen in the overview publication [26].

### Study design

The study was conducted between October 2007 and September 2013 as a prospective case-control multi-centre questionnaire study. Following inclusion on the national research network portfolio (NIHR CRN) in 2012, study recruitment increased to an average of 100 participants per month with a recruitment rate of 66%. The study specific questionnaire was anonymous and therefore no consent was required, but implied through participation. Participant information leaflets were provided. Questionnaires were either completed before leaving the clinic or taken home and returned by post in prepaid envelopes. The returned questionnaires were then scanned into a database electronically, and the electronic records were then checked by two members of the research team for accurate correlation with the paper questionnaires and for missing data. The questionnaire was completed on one occasion only.

### Setting

A total of 30 secondary/tertiary care sites widely spread across the UK including the devolved nations of Wales and Scotland participated in the study where general otorhinolaryngology or subspecialist rhinology clinics managed patients referred from primary care.

### Participants

Patients were recruited at the point of contact during outpatient consultation in secondary care, regardless of prior management in either primary or secondary care and regardless of prior surgical intervention. They were classified by sub group of CRS (CRSsNPs, CRSwNPs or AFRS) by a clinician on the basis of their history and endoscopic and/or CT findings, prior to completion of the questionnaire. Controls who had no diagnosis of nasal or sinus conditions were recruited from amongst hospital staff and also family and friends of those attending ENT outpatient clinics (regardless of cause), provided they met the criteria below.

### Inclusion criteria

Criteria for diagnosis of chronic rhinosinusitis (CRS) with or without polyps (EPOS guidelines) [1]:

Symptoms must be present for at least 12 weeks and include:

- nasal blockage/obstruction/congestion and/or nasal discharge (anterior/posterior nasal drip)
- and either facial pain/pressure and/or reduction or loss of sense of smell

and additionally:

- endoscopic signs of: polyps and/or mucopurulent discharge primarily from middle meatus and/or; oedema/mucosal obstruction primarily in middle meatus
- and/or CT changes: mucosal changes within the ostiomeatal complex and/or sinuses

Patients classified as AFRS adhered to either the Bent and Kuhn criteria (see above) or the modified Vancouver criteria [27].

### Exclusion criteria

- Patients unable to comprehend written English.
- Patients under the age of 18 years.

### Exclusion criteria for the control group

- Active sinonasal disease - e.g. ARS, CRS, rhinitis
- Medical co-morbidity being actively treated
- Hospitalisation within the last 12 months

### Variables and data sources

The study questionnaire included various specific questions for allergy and asthma as follows:

*“Do you have any known confirmed allergies (on a skin prick or blood test) e.g house dust mite? Yes/No”* followed by a free text box asking participants to state any allergies.

*“Do you have any suspected allergies? Yes/No”*, also followed by a free text box.

*“Have you ever experienced any allergy symptoms such as wheezing, runny nose or itchy skin when taking aspirin?* Yes/No”.

And under the topic of medical comorbidities, *“Do you have any of the following medical problems?: Asthma…”.*

Participants did not separately undergo skin prick tests or RAST inhalant screens as part of the study.

### Bias

All of the comorbid conditions assessed were based on self-reporting but the questionnaire design and subsequent analysis was such that the impact of this has been minimized and will be equal across all groups. Aspirin sensitivity was determined by asking specifically about responses to aspirin that affect respiratory mucosa, such as wheezing and rhinorrhoea, so that those with only gastrointestinal intolerance should not define themselves as aspirin allergic for the purposes of this questionnaire. NSAID (non-steroidal anti-inflammatory drug) allergy was not specifically enquired about but a free text box was included for any additional allergies. Both asthma diagnosis and aspirin sensitivity are therefore self-reported, but the former was additionally correlated with reported asthma medication.

### Sample size calculation

The sample size calculation was based on the primary outcome of the study which was to look for common associations between socioeconomic factors and CRS. For socio−economic scores, the standard approach is to compare the proportion of subjects in the lower social classes to everyone else. In order for the study to have 80% power to detect a difference of 10% in “low social class” between controls and CRS patients, assuming a 30% rate in the CRS patients, with approximately 5 CRS patients to 1 control patient, 965 CRS patients and 193 controls were required [26].

### Statistical analysis

For the purposes of these analyses we have used descriptive statistics to describe the sample; differences in the rates of asthma, aspirin sensitivity and inhalant allergy between groups were assessed by Chi-Squared tests. For the analysis of aspirin sensitivity, in order to account for the potential confounding effects, logistic regression was also used, firstly, we adjusted the analysis for aspirin sensitivity and secondly we adjusted for asthma diagnosis, age and gender.

## Results

### Participant flow and missing data

**Fig. 1:**
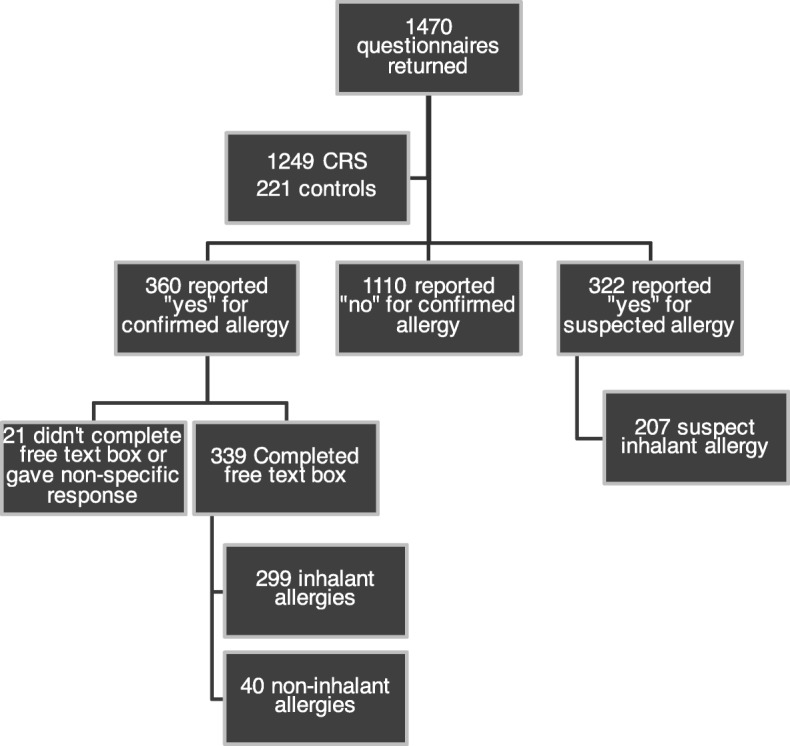
Participant Flow

Participants with allergies that were self-reported as having been previously confirmed by skin prick test or RAST, and those reporting suspected allergies were included in this analysis. Figure 1 shows the details including 21 participants who did not complete the free text box.

### Descriptive data

A total of 1470 participants’ questionnaires were available for analysis; 1249 with CRS (CRSsNP 553, CRSwNP 651, AFRS 45) and 221 controls. The age range was 17–102 years (mean 52) with 54% reported as male (Table 1).

**Table 1:** Demographics of the participants

	Controls	CRSsNPs	CRSwNPs	AFRS
Participants	221	553	651	45
Females (%)	143 (68.4%)	259 (53.1%)	185 (32.2%)	19 (43.2%)
Mean Age (SD)	47.3 (14.9)	51.8 (15.3)	56.0 (14.6)	56.1 (12.7)
Age Range	19–82	18–84	17–102	20–76
Asthma (%)	22 (9.95)	117 (21.16)	303(46.90)	33 (73.33)
Aspirin Sensitivity (%)	5 (2.26)	18 (3.25)	62 (9.61)	18 (40.0)

### Main results

#### Asthma

Those with CRS were more likely to suffer from asthma, with those with nasal polyp subtypes even more likely to report asthma including the majority of those with AFRS (see Table 1). Prevalence of asthma ranged from 21% in CRSsNPs through 47% in CRSwNPs to 73% in AFRS. Those with self-reported confirmed inhalant allergy and asthma (“allergic asthmatics”) are detailed below.

#### Aspirin sensitivity

Those with CRS were more likely to report aspirin sensitivity. In a similar manner to asthma, those with nasal polyp subtypes were increasingly likely to report aspirin sensitivity (AFRS more so than CRSwNPs); odds ratio 28.8 (CIs 9.89–83.8). The odds ratio for aspirin sensitivity after adjustment for asthma diagnosis showed that only those with AFRS were significantly more likely to report aspirin sensitivity (OR 9.64, *p* < 0.001). There was no significant interaction between CRS type and asthma on the odds of aspirin sensitivity; this indicates that aspirin sensitivity status is influenced by both asthma diagnosis and CRS group independently. There were no significant differences between males and females (Table 2).

**Table 2:** Odds ratio of aspirin sensitivity by diagnosis, unadjusted and adjusted analysis

	Factor	Total number	Asthma (%)	Frequency of Aspirin Sensitivity (%)	Odds Ratio (OR)(95% CI)	p-value	Adjusted OR (95%CI)^a^	*p*-value	Adjusted+ 2 OR (95%CI)^b^	p-value for OR
Group	Control	221	22 (9.95)	5 (2.26)	1		1		1	
	CRSsNP	553	117 (21.16)	18 (3.25)	1.45 (0.53, 3.96)	0.465	1.03 (0.37, 2.88)	0.948	1.08 (0.34,3.4)2	0.893
	CRSwNP	651	303 (46.90)	62 (9.61)	4.59 (1.82, 11.58)	**< 0.001**	2.00 (0.76, 5.25)	0.158	2.39 (0.80,7.18)	0.120
	AFRS	45	33 (73.33)	18 (40.0)	28.8 (9.89, 83.8)	**< 0.001**	9.61 (3.12, 29.63)	**< 0.001**	**12.20 (3.49,42.68)**	**< 0.001**
Asthma	No	968		21 (2.12)						
	Yes	392		82 (17.3)	9.64 (5.89,15.79)	**< 0.001**				

^a^Adjusted for asthma diagnosis

^b^Adjusted for asthma diagnosis, age and gender

#### Inhalant allergy

**Fig. 2:**
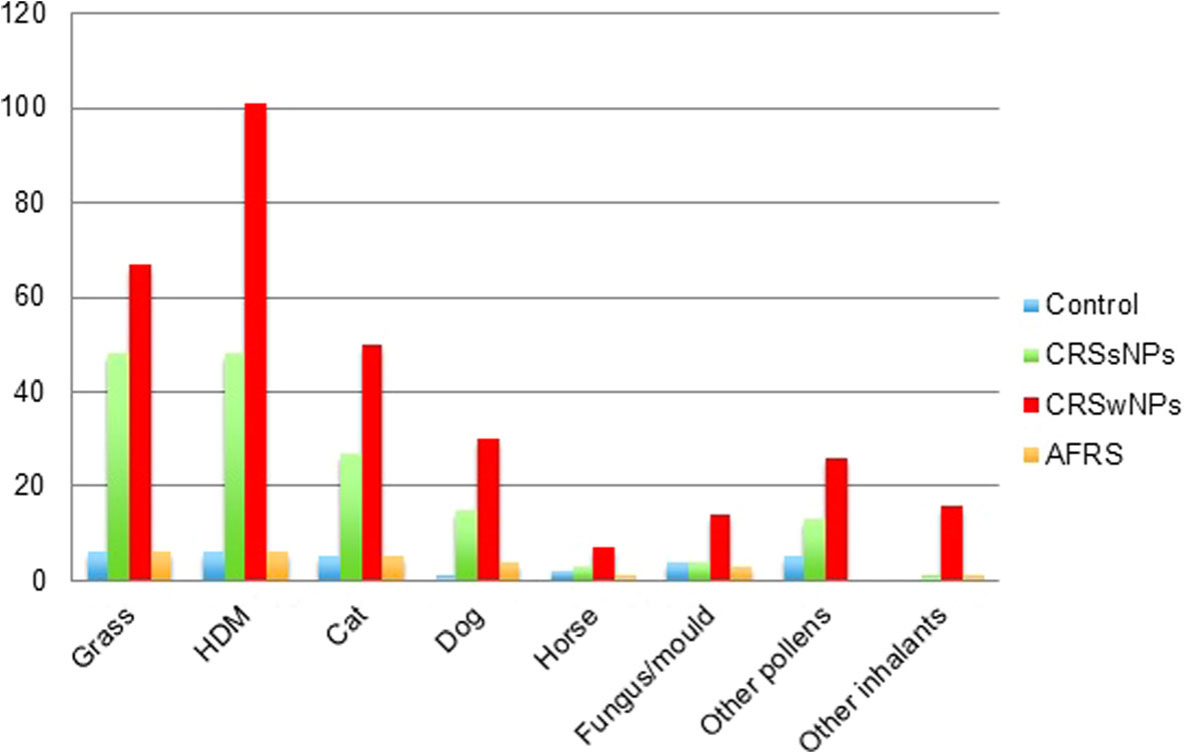
Frequency of confirmed inhalant allergy in CRS participants

Patients with CRS were also significantly more likely to report having a confirmed inhalant allergy (via skin prick test (SPT) or RAST) than controls. Once again the pattern mirrored that for asthma and aspirin sensitivity above, with 1 in 5 CRSsNPs and 1 in 3 CRSwNPs/AFRS cases reporting inhalant allergies (*p* < 0.02, Table 3). The most commonly reported confirmed inhalant allergy was house dust mite followed by grass pollen (Table 4, Fig. 2). The prevalence of house dust mite allergy was significantly higher in the CRSwNPs group compared to the CRSsNPs group (*p* < 0.001). Furthermore 356 participants reported suspected allergy (i.e. not confirmed by SPT/RAST) of which 228 reported sensitivity to inhalant allergens (207 with CRS, 21 controls). Asthmatics with self-reported “confirmed” inhalant allergy (Table 5) are proportionally consistent across the groups but viewed as a percentage of each group are seen to be most prevalent in the CRSwNPs and AFRS groups at 20 and 29% respectively; odds ratios expressed for “allergic asthmatics” were 6.71 and 10.82 within the CRSwNPs and AFRS groups respectively.

**Table 3:** Inhalant allergy by subgroup (vs control)

	Frequency of confirmed inhalant allergy	%	Percentage difference compared to controls	Odds ratio (vs control) 95% Confidence interval	p-value
Control	29	13.1	1	N/A	N/A
CRSsNPs	113	20.3	7.2	1.70 (1.09,2.64)	0.019
CRSwNPs	203	31.0	17.9	3.03 (1.98,4.63)	< 0.001
AFRS	15	33.3	19.9	3.31 (1.59,6.89)	0.001

**Table 4:** Confirmed and suspected inhalant allergens

			Grass	HDM	Cat	Dog	Horse	Fungus/mould	Other pollens	Other inhalants	Number of participants with inhalant allergies
1	Self-reported RAST/SPT confirmed allergies	Control	6	6	5	1	2	4	5	0	16
		CRSsNPs	48	48	27	15	3	4	13	1	93
		CRSwNPs	67	101	50	30	7	14	26	16	177
		AFRS	6	6	5	4	1	3	0	1	13
2	Suspected allergies	Control	4	4	7	1	1	2	4	0	21
		CRSsNPs	18	22	7	4	1	7	29	3	95
		CRSwNPs	15	28	12	5	0	6	26	2	106
		AFRS	1	2	0	0	0	2	1	0	6
3	Combined confirmed and suspected allergies	Control	10	10	12	2	3	6	9	0	37
		CRSsNPs	66	70	34	19	4	11	42	4	188
		CRSwNPs	82	129	62	35	7	20	52	18	283
		AFRS	7	8	5	4	1	5	1	1	19
Chi-Squared test (*or Fisher exact*) p-value		1	0.395	< 0.001	0.063	0.115	0.358	0.073	1	0.002	
		2	0.407	0.893	0.569	1	n/a	0.767	0.37	0.855	
		3	0.795	0.001	0.041	0.138	0.737	0.37	0.884	0.016

**Table 5:** Frequency of participants with self-reported “confirmed” inhalant allergy and asthma

Group	Frequency	Percentage (of asthmatics in group)	Percentage of whole group	OR (95% CI) (whole group)	OR (95% CI) (asthmatics)
Controls	8	28.9	3.6		
CRSsNPs	42	27	7.6	2.19 (1.01,4.74)	0.98 (0.38,2.53)
CRSwNPs	130	37	20.1	6.71 (3.22,13.94)	1.32 (0.54,3.23)
AFRS	13	33	28.9	10.82 (4.16,28.13)	1.14 (0.37,3.47)

## Discussion

### Key results

Our study has shown a significantly higher prevalence of asthma, inhalant allergy and aspirin sensitivity within the phenotypes of CRS where nasal polyps are present. This reflects the substantial interaction between the lower and upper airways and in particular between the underlying aetiological mechanisms of airways pathology. Similar interaction is also found in those with allergic rhinitis, and has been extensively reported by the ARIA (Allergic Rhinitis and its Impact on Asthma) Taskforce [28]. Those in the AFRS subgroup have a very high prevalence of both asthma and aspirin sensitivity that could indicate an overlap between AFRS and what may be AERD. The prevalence of allergy within this large national sample of CRS patients at 26% of all CRS cases is towards the lower end of the range of allergy reported in the literature as mentioned above, lending weight to allergy as an associative factor in CRS rather than an aetiological factor.

### Strengths and limitations

The study is a large cross-sectional study including a varied population from across the United Kingdom. It is the largest epidemiological study of CRS in the UK to date. In contrast to other epidemiological studies in CRS, patients recruited were diagnosed by an otorhinolaryngologist in keeping with international guidelines. According to Asthma UK, the prevalence of asthma in adults in the UK is 1 in 12 or 8.3%, a similar number to our control population.

A weakness of the study is that, with the exception of the diagnosis of CRS, it relies on participants’ self-reported information, rather than actual skin prick test/RAST results or an aspirin provocation test. However, the questionnaire was worded to be as explicit as possible so that participants were likely to pick the most accurate option, for example, the question regarding aspirin allergy is phrased so as to identify respiratory and nasal-type allergy symptoms rather than gastrointestinal disturbances. Any potential error in self-reporting or recall should be equal across CRS groups so should not bias the results as far as comparison between subgroups. It is not intended that these results be used as a prevalence study for either condition amongst the general population, but they can show prevalence of both aspirin allergy and asthma in a large cohort of CRS patients. Additionally, controls had no self-reported nasal symptoms but did not undergo nasal examination. Similarly, the reporting of confirmed inhalant allergy testing is also prone to recall bias but again this is likely to be equal across all of the groups compared.

Despite clear criteria for the diagnosis of AFRS, some patients with nasal polyps who have AERD could have been erroneously categorised in the AFRS group rather than the CRSwNPs group by clinicians. Conversely, diagnosis of AFRS requires vigilance and careful investigation by clinicians, but is also limited by local laboratory facilities, so in this multicentre study it is likely that some patients in the CRSwNPs category will in fact have undiagnosed AFRS. Consequently, the association between AFRS and aspirin sensitivity/asthma may be even stronger than has so far been described. Another caveat is that RAST results do require clinical correlation too [29].

### Interpretation

CRS is known to be a complex spectrum of disease associated with respiratory co-morbidities. Basic phenotypes are currently recognised and we have shown their differing associations with asthma and allergies; such phenotypes are likely to be refined over time with new definitions that may reflect these results and include the presence or absence of concomitant allergy or allergic response. Evidence shows that the actual rate of aspirin sensitivity is higher than the patient-reported rate [30]. Had we been able to provide an aspirin provocation test for this cohort, then the study by Szczeklik et al. suggests we would have discovered an additional 15% of cases who were unaware of this sensitivity.

Those with AFRS were most likely to report sensitivity to aspirin. In AERD, the pathophysiology includes changes in the metabolism of arachidonic acid, release of inflammatory mediators and cytokines, and involvement of microorganisms including bacteria and viruses [31]. Abnormal metabolism of arachidonic acid is characterized by an imbalance between cyclooxygenase (COX) and lipoxygenase pathways that results in an overactive lipoxygenase pathway. This is accentuated with aspirin and non-steroidal drug ingestion in susceptible patients, leading to increased production of leukotrienes and intensification of airway inflammation. A similar inflammatory mechanism might explain the increased sensitivity to aspirin experienced by those with AFRS. Elevated release of inflammatory mediators, such as histamine, have also been found to be elevated in those suffering from CRSwNPs and aspirin The majority of patients with AERD are thought to develop nasal polyps during the course of their disease [31]. Their polyposis tends to be more extensive and difficult to treat medically, as well as presenting with higher recurrence rates after surgery, in a similar manner to those with AFRS [8], therefore crossover of diagnoses are a strong possibility. Nasal tissue biopsy specimens from patients with AERD have shown infiltration of eosinophils and degranulated mast cells. AERD is an acquired disorder and aspirin hypersensitivity can occur in patients who already have chronic or allergic rhinitis and asthma [30]. The link between AERD and preceding allergic rhinitis has been suggested [32] and a significant number of patients who develop AERD report preceding symptomatic inhalant allergy [33] but a definitive link remains elusive.

The evidence presented here supports that from a smaller study of 51 patients from the Mayo clinic in 1994 [34], with our reported prevalence of both asthma and aspirin sensitivity of 58.8 and 29.0% in the AFRS cohort comparable with their results of 54 and 27% respectively. A much smaller Malaysian study reported a prevalence of asthma and aspirin sensitivity as 37.5 and 25% respectively [35]. Our study is the largest to consider a spectrum of CRS disorders as well aspirin sensitivity and asthma diagnoses. AERD has been found to affect 0.3–2.5% of the general population [31], a similar figure to the number of participants who reported aspirin sensitivity amongst our control and CRSsNPs groups (3 and 4.2% respectively). This increased prevalence of aspirin sensitivity combined with typically more aggressive inflammatory disease amongst those with AFRS may therefore reflect a more complex or separate pathophysiological process leading to its development [36].

### Generalisability

Phenotypes of CRS with nasal polyps were associated with an increased prevalence of aspirin sensitivity and inhalant allergies, and therefore we hypothesise that, clinically, this consideration may be helpful in the early identification of patients who are more likely to suffer from combined airway disease, both in primary and secondary care. The diagnosis of concurrent AR should also be considered in all patients with CRS, and focused history taking should alert clinicians to the need for formal allergy testing.

Treatment of rhinitis is thought to reduce asthma severity, so prompt treatment has an impact on both upper and lower respiratory tract symptoms [37]. Patients themselves report experiencing upper and lower respiratory symptoms which exacerbate each other. Care, however, is normally very divided between ENT and Respiratory medicine with separate clinic teams for upper and lower respiratory symptoms. In the UK, dedicated allergists are still only few in number, and many patients with allergies will never consult directly with an allergist. Patients report difficulty in accessing care which takes both upper and lower respiratory symptoms into account, and this should be considered, with combined clinics or close working relationships likely to improve quality and efficiency of care [38]. Patients with asthma and/or allergies have been found to be more likely to experience delayed surgical intervention, and delayed surgical intervention itself has been found to lead to less improvement in symptoms than early surgery [39]. Patients with asthma and aspirin or inhalant allergies may therefore benefit from more aggressive treatment, including timelier referral to specialist services and a united approach from the clinicians involved.

Patients with AERD are more likely to suffer from allergies in general [8], and it may be important to consider testing for such allergies more comprehensively. Desensitisation might be considered in patients with severe aspirin or inhaled allergies. The current diagnostic criteria for AFRS [4] do not take aspirin sensitivity into account, but our results suggest that as aspirin sensitivity occurs in 40% of patients with AFRS, consideration should be given to including it amongst the minor criteria along with asthma, Charcot-Leyden crystals and peripheral eosinophilia. Patients with both asthma and CRSwNPs may also derive benefit from leukotriene receptor antagonists and may be as beneficial as INCS [40].

## Conclusion

The prevalence of asthma and allergy in CRS varies by phenoytype with CRSwNPs and AFRS having a stronger association with both. Aspirin exacerbated respiratory disease has a large overlap with allergic fungal rhinosinusitis suggesting some common pathophysiology. These comorbidities are significantly more prevalent than in non-CRS controls and strengthen the need for a combined airways approach to inflammatory respiratory tract disease, with particular attention to assessment of allergy status. Large-scale studies with standardised objective assessment of allergy status would help to unravel any shared pathophysiology between these diseases and could guide more efficient management. Longitudinal studies to look at the natural history of CRS may also to understand the role of allergy and aspirin sensitivity in relation to the pathophysiology.
